# Ichthyosis Uteri Mimicking Endometrial Cancer with Apparent Myometrial Invasion

**DOI:** 10.31662/jmaj.2025-0032

**Published:** 2025-08-01

**Authors:** Mikiya Fujii, Kota Yokoyama, Akiko Suzuki, Eisaku Ito, Sho Murakami, Mayuko Tanaka, Shinichi Taura, Ukihide Tateishi

**Affiliations:** 1 Department of Diagnostic Radiology, Ome Medical Center, Tokyo, Japan; 2 Department of Radiology, Institute of Science Tokyo, Tokyo, Japan; 3 Department of Obstetrics and Gynecology, Ome Medical Center, Tokyo, Japan; 4 Department of Pathology, Ome Medical Center, Tokyo, Japan

**Keywords:** ichthyosis uteri, squamous metaplasia, endometrial cancer, adenomyosis, magnetic resonance imaging

## Abstract

Ichthyosis uteri is a rare benign condition characterized by extensive squamous metaplasia of the endometrium. We present an unusual case of ichthyosis uteri with radiological features mimicking myometrial invasion of endometrial cancer, occurring in a 50-year-old postmenopausal woman who presented with persistent abnormal vaginal bleeding. Laboratory findings revealed elevated serum squamous cell carcinoma (SCC) antigen level of 11.5 ng/mL. Magnetic resonance imaging (MRI) demonstrated an intrauterine lesion with apparent myometrial invasion, initially suggesting the International Federation of Gynecology and Obstetrics stage IA endometrial cancer. The lesion showed low signal intensity on the T2-weighted image compared to normal endometrium and weak enhancement on the contrast-enhanced T1-weighted image. While the lesion showed high signal intensity on diffusion-weighted imaging, the apparent diffusion coefficient value (0.96 × 10^-3^ mm^2^/sec) indicated no substantial diffusion restriction. Despite the biopsy showing only squamous metaplasia without malignancy, a total hysterectomy was performed due to persistent bleeding, elevated serum SCC antigen level, and suspected myometrial invasion. Pathological examination revealed extensive papillary proliferation of bland squamous epithelium extending along adenomyosis, with concurrent endometrial hyperplasia. Postoperative serum SCC antigen level normalized to 0.7 ng/mL. This case highlights a unique radiological presentation of ichthyosis uteri, where extension along adenomyotic foci mimicked myometrial invasion of endometrial cancer. The relative lack of diffusion restriction on MRI, despite the lesion’s size, may serve as a valuable diagnostic clue in differentiating ichthyosis uteri from endometrial cancer.

## Introduction

Ichthyosis uteri represent an uncommon pathological entity characterized by extensive squamous metaplasia of the endometrium ^(1)^, with a predilection for postmenopausal women ^(2)^. While the condition itself is benign, its clinical and radiological presentation can mimic endometrial malignancy, potentially leading to diagnostic challenges. Although several cases of ichthyosis uteri have been reported in the literature, detailed imaging findings, particularly in cases with concurrent adenomyosis, remain poorly documented. Here, we present a case of ichthyosis uteri that radiologically mimicked endometrial cancer due to its extension pattern along adenomyosis.

## Case Report

A 50-year-old postmenopausal woman presented with abnormal vaginal bleeding persisting for three months. Her medical history was unremarkable except for a previous cesarean section. Laboratory evaluation revealed an elevated serum squamous cell carcinoma (SCC) antigen level of 11.5 ng/mL (reference range <1.5 ng/mL).

**Figure 1.**
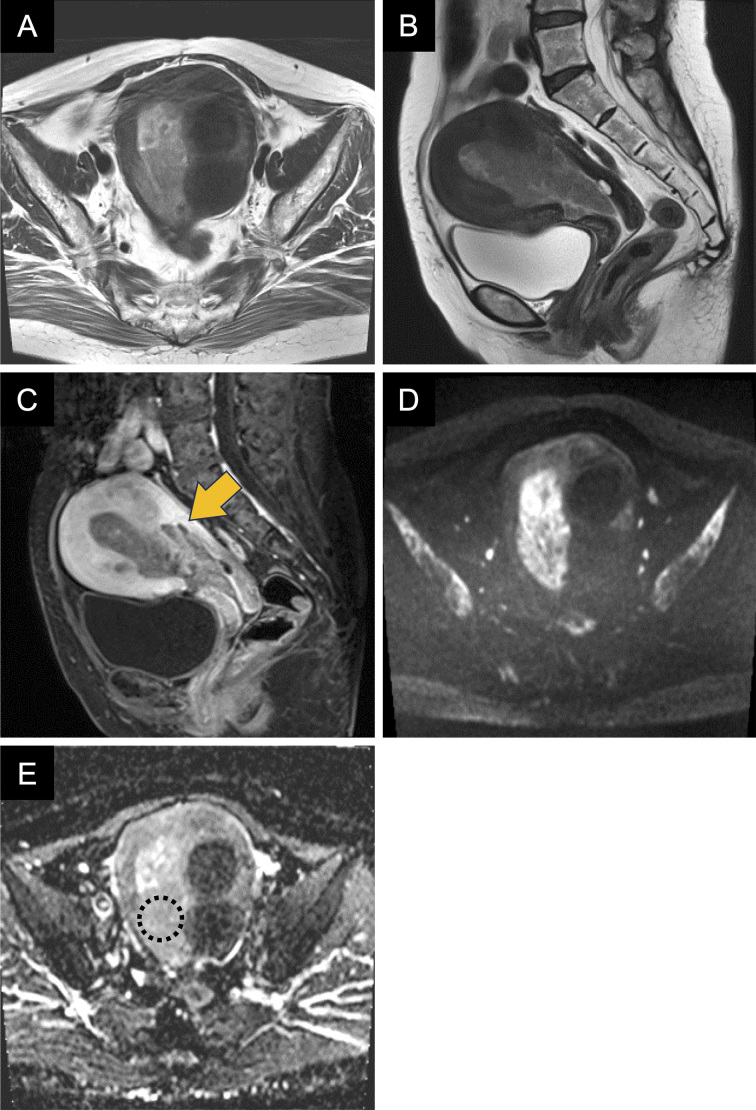
MRI findings of endometrial lesion. The lesion shows low signal intensity on the T2-weighted image compared to normal endometrium (A, B). There were no findings suggestive of adenomyosis. On contrast-enhanced T1-weighted image, the lesion demonstrates weak enhancement with suspected invasion into the posterior wall of the uterine corpus (C; arrow). While the lesion shows high signal intensity on DWI (D), the ADC value of 0.96 × 10^-3^ mm^2^/sec indicates no substantial diffusion restriction (E; dotted circle). Multiple uterine leiomyomas were observed. ADC: apparent diffusion coefficient; DWI: diffusion-weighted imaging; MRI: magnetic resonance imaging.

Magnetic resonance imaging (MRI) revealed an intrauterine lesion showing low signal intensity on the T2-weighted image compared to normal endometrium (Figure 1A and B) with weak enhancement on the contrast-enhanced T1-weighted image (Figure 1C). The lesion appeared to invade the posterior myometrium (Figure 1C; arrow). While the lesion showed high signal intensity on diffusion-weighted imaging (Figure 1D), the apparent diffusion coefficient (ADC) value was 0.96 × 10^-3^ mm^2^/sec, indicating no substantial diffusion restriction (Figure 1E; dotted circle). There were no findings suggestive of adenomyosis. With suspected superficial myometrial invasion, the International Federation of Gynecology and Obstetrics stage IA endometrial cancer was considered. Despite the biopsy showing only squamous metaplasia without malignancy, total hysterectomy with bilateral salpingo-oophorectomy was performed due to persistent bleeding, elevated serum SCC antigen level, and suspected myometrial invasion.

**Figure 2.**
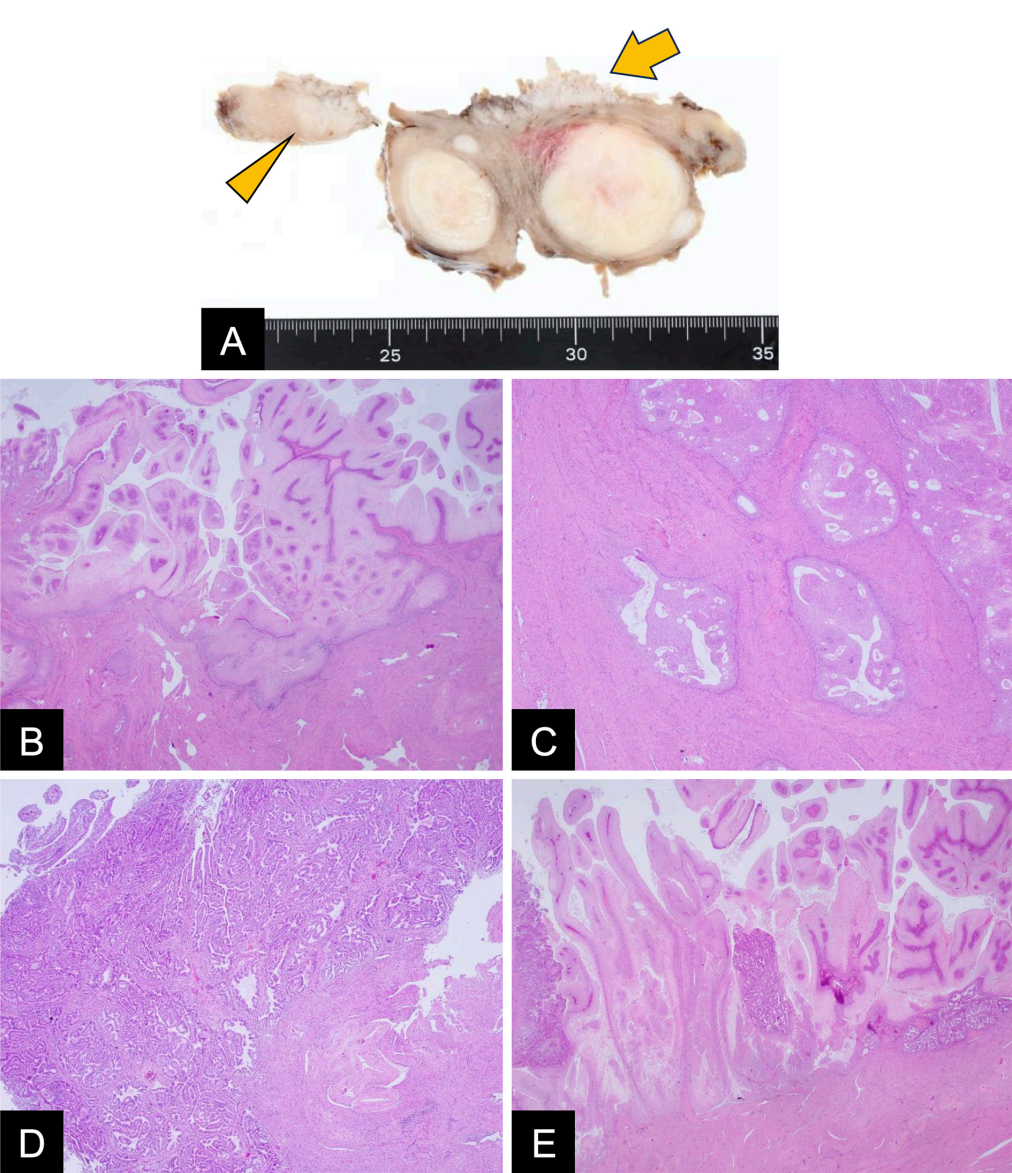
Gross and microscopic pathology findings. Gross examination revealed a white papillary lesion on the endometrial surface (A; arrow) with focal myometrial extension (A; arrowhead). Microscopically, extensive papillary proliferation of bland squamous epithelium was observed (B), which extended 15 mm along adenomyosis (C). Residual glands showed hyperplasia (D), with areas showing mixed squamous metaplasia and hyperplastic changes (E).

Gross examination revealed a white papillary lesion on the endometrial surface (Figure 2A; arrow) with focal myometrial extension (Figure 2A; arrowhead). Microscopically, extensive papillary proliferation of bland squamous epithelium was observed (Figure 2B), which extended 15 mm along adenomyosis (Figure 2C). Residual endometrial glands showed hyperplasia (Figure 2D), with areas showing mixed squamous metaplasia and hyperplastic changes (Figure 2E). Immunohistochemistry was negative for p16. The cervix and adnexa were unremarkable. Postoperative serum SCC antigen level normalized to 0.7 ng/mL.

## Discussion

Ichthyosis uteri is a rare condition characterized by extensive squamous metaplasia replacing the endometrium, predominantly affecting postmenopausal women. Although ichthyosis uteri are generally considered benign, previous reports have documented cases with concurrent endometrial adenocarcinoma ^(3), (4)^ or SCC ^(5), (6)^, highlighting the importance of careful evaluation. The etiology of ichthyosis uteri remains incompletely understood, although chronic inflammation has been implicated in previous reports ^(3), (7), (8)^; however, no clinical or histological evidence of inflammation was observed in the present case. While the precise etiology remains unclear, the concurrent presence of endometrial hyperplasia might suggest a potential underlying relationship between these conditions.

The most striking feature of this case was the MRI appearance of myometrial invasion mimicking endometrial cancer. The radiological-pathological correlation in the present case revealed that the appearance of myometrial invasion was attributable to the extension of squamous metaplasia along adenomyotic foci, representing a novel diagnostic pitfall in the radiological assessment of endometrial pathology. In this case, the MRI findings shared several characteristics with endometrial cancer, particularly the hypo-enhancing lesion with apparent myometrial invasion. However, the relative lack of diffusion restriction despite the substantial size of the lesion provided a valuable diagnostic clue for differentiation from endometrial cancer. The observed ADC value of 0.96 × 10^-3^ mm^2^/sec, while potentially overlapping with some cases, was relatively higher than typical values reported for endometrial carcinoma ^(9)^, providing a crucial quantitative parameter for differential diagnosis.

The elevated serum SCC antigen level in this patient is noteworthy. While serum SCC antigen is typically elevated in SCCs, such as cervical carcinoma ^(10)^, no malignant squamous lesions were detected in the patient. The normalization of serum SCC antigen level following surgical intervention suggests that the elevated preoperative level was likely attributable to the extensive squamous metaplasia of ichthyosis uteri.

In conclusion, this report presents a diagnostically challenging case of ichthyosis uteri that radiologically mimicked myometrial invasion of endometrial cancer in the setting of concurrent adenomyosis. The combination of elevated serum SCC antigen level and apparent myometrial invasion on MRI initially raised concern for malignancy. However, the relatively high ADC values on MRI provided a valuable diagnostic clue for differentiating this benign entity from endometrial cancer. Recognition of this distinctive pattern of ichthyosis uteri extending along adenomyotic foci is crucial for appropriate clinical management. Comprehensive assessment integrating radiological, histopathological, and biochemical findings is essential for accurate diagnosis and optimal therapeutic decision-making in this diagnostically challenging case.

## Article Information

### Conflicts of Interest

None

### Author Contributions

Mikiya Fujii, Kota Yokoyama, Akiko Suzuki, Eisaku Ito, Sho Murakami, Mayuko Tanaka, Shinichi Taura, and Ukihide Tateishi contributed to the acquisition, analysis and interpretation of data. Mikiya Fujii prepared the initial draft of the manuscript. All authors critically revised the manuscript and approved the final version for submission.

### Approval by Institutional Review Board (IRB)

Not applicable.

### Informed Consent

Informed consent was obtained from the patient.

## References

[1] Patton WT, Squires GV. Icthyosis uteri. A case report. Am J Obstet Gynecol. 1962;84(7):858-60. 13941928 10.1016/0002-9378(62)90060-1

[2] Zhang Y, Tounsi S, Yadav G, et al. Ichthyosis uteri: a keratinizing squamous metaplasia of the endometrium with premalignant potential. Gynecol Oncol Rep. 2023;46:101165. 36968297 10.1016/j.gore.2023.101165 PMC10033720

[3] Shi H, Chen X, Zhang S, et al. Ichthyosis uteri complicated by poorly differentiated endometrial adenocarcinoma with squamous differentiation. Prz Menopauzalny. 2013;17(6):449-52.

[4] Bewtra C, Xie QM, Hunter WJ, et al. Ichthyosis uteri: a case report and review of the literature. Arch Pathol Lab Med. 2005;129(5):e124-5. 15859657 10.5858/2005-129-e124-IUACRA

[5] Murhekar K, Majhi U, Sridevi V, et al. Does “ichthyosis uteri” have malignant potential? A case report of squamous cell carcinoma of endometrium associated with extensive ichthyosis uteri. Diagn Pathol. 2008;3:4. 18234121 10.1186/1746-1596-3-4 PMC2259312

[6] Takeuchi K, Tsujino T, Yabuta M, et al. A case of primary squamous cell carcinoma of the endometrium associated with extensive “ichthyosis uteri.” Eur J Gynaecol Oncol. 2012;33(5):552-4. 23185812

[7] Bhardwaj N, Diwaker P, Gogoi P, et al. Ichthyosis uteri associated with endometrial adenocarcinoma: A case report. J Clin Diagn Res. 2017;11(6):ED24-5. 10.7860/JCDR/2017/27951.10116 PMC5535377 28764184

[8] Kanno K, Kusakabe T, Takata M, et al. Ichthyosis uteri with leiomyoma. J Obstet Gynaecol Res. 2016;42(11):1599-603. 27528500 10.1111/jog.13108

[9] Rechichi G, Galimberti S, Signorelli M, et al. Endometrial cancer: correlation of apparent diffusion coefficient with tumor grade, depth of myometrial invasion, and presence of lymph node metastases. AJR Am J Roentgenol. 2011;197(1):256-62. 21701038 10.2214/AJR.10.5584

[10] Bolli JA, Doering DL, Bosscher JR, et al. Squamous cell carcinoma antigen: clinical utility in squamous cell carcinoma of the uterine cervix. Gynecol Oncol. 1994;55(2):169-73. 7959279 10.1006/gyno.1994.1272

